# Neuropsychology of the parietal lobe: Luria’s and contemporary conceptions

**DOI:** 10.3389/fnins.2023.1226226

**Published:** 2023-10-20

**Authors:** Dyecika Souza-Couto, Rafael Bretas, Tales Alexandre Aversi-Ferreira

**Affiliations:** ^1^Laboratory of Biomathematics and Physical Anthropology, Federal University of Alfenas, Alfenas, Brazil; ^2^Innovation Design Office, Riken, Kobe, Japan

**Keywords:** parietal lobe, neuropsychology, functions of the parietal lobe, second functional unit, posterior parietal cortex

## Abstract

The parietal lobe, constituting approximately 20% of the human brain, comprises two main regions: the somatosensory cortex and the posterior parietal cortex. The former is responsible for receiving and processing information from the organism itself or its external environment, while the latter performs concurrent summaries and higher cognitive functions. The present study seeks to integrate modern research findings with Luria’s previous discoveries in order to gain a nuanced understanding of the roles assigned to the parietal lobe as well as its lateralization differences.

## 1. Introduction

The socio-historical-cultural theory is a psychological movement that emerged in Russia in the early 20th century, and it is based on the work of Vygotsky (1896–1934). The theory has been widely adopted by Brazilian psychologists and educators to inform various discussions on the philosophy of education. In this context, the theory aims to explain some aspects of the mind by considering the social, historical, and cultural influences on human development (Oliveira and Rego, 2010; Aversi-Ferreira et al., 2019).

One of the main proponents of this theory was Luria (1902–1977), a neuropsychologist who continued and expanded upon Vygotsky’s legacy after his premature death at the age of 37 (Aversi-Ferreira et al., 2019). Luria’s work explored the relationship between brain functions and mental processes using clinical observations, experimental methods, and cross-cultural studies (Luria, 1979, 1996), for which he combined his training in medicine with his academic career in psychology (Aversi-Ferreira et al., 2010).

Luria challenged the then-dominant view that brain functions were strictly localized in specific regions. This view was mainly based on the discoveries of Broca and Wernicke, who identified brain areas related to language production and comprehension. However, Luria argued that their approach was too simplistic and ignored the complex interactions among different brain structures and neural pathways. Moreover, at that time, knowledge of the cortical cytoarchitecture and neural pathways was still scarce (Luria et al., 1970; Luria, 1973).

Overall, Luria aimed for a deeper understanding of the brain, which, according to the historical-social-cultural theory, required knowledge from studies in social science, phylogenetics, anthropology, and neurophysiology related to human and animal brains and behavior (Luria, 1979, 1996). However, before he could collate the data from these different fields in a logical and complex interdisciplinary way, he needed to elucidate several complex brain functions that at that time were not well understood.

To this end, he began studying patients with local brain lesions and developed a method of analysis to evaluate the functions that were impaired by the lesions (Luria and Rapoport, 1962; Luria, 1971, 1973, 1987b). This process was difficult (for more details see Luria, 1987a), because brain areas do not have unique, specifically defined functions. Instead, each structure works together with others (Luria, 1973), so between two patients the same structure may fail differently due to differences in the presence of other damaged areas (Luria, 1987b).

Although Luria’s work was vital to the development of the field of neuropsychology, some more recent research has diverted from his original focus. The Cold War played a role in preventing certain parts of Luria’s research from being disseminated to the West, resulting in contemporary papers on neuropsychology frequently drawing on Luria’s work without explicitly referencing it (Aversi-Ferreira et al., 2010, 2019). However, recent efforts have been made to increase the visibility of Luria’s studies and hypotheses among psychologists and psychiatrists, particularly through citations in papers.

According to the book The Working Brain – An Introduction to Neuropsychology (Luria, 1973), Luria proposed the existence of three functional units to explain, in general, the brain’s workings. These units were associated with specific structures in the brain, with the first unit’s main structure linked to the midbrain and the reticular formation, the second unit’s main location placed in the posterior inferior region of the parietal lobe, and the third unit linked to the frontal lobe. According to Luria’s theories, the second unit is responsible for the reception, analysis, and storage of information, making it the receptor region of the brain. However, while the parietal lobe plays an important role as a receptor station of the cortex, it is also involved in complex behaviors.

Recent studies have examined the relationship between Luria’s neuropsychology and current discoveries in the field. One such study evaluated the effectiveness of Luria’s work by likening his study of the parietal lobe and its association with neuropsychology to current neurophysiology (Aversi-Ferreira et al., 2010) but with a more structural focus. The study concluded that while advancements in image diagnostics may generate more information for neuropsychology, the concepts of temporal neuropsychology have not undergone significant modifications (Aversi-Ferreira et al., 2019).

## 2. Materials and methods

In this work, we propose a systematic methodology to seek articles published after 1973 concerned with the neurophysiology of the parietal lobe, because in 1973 the first edition of the book The Working Brain – An Introduction To Neuropsychology (Luria, 1973) was published in English, which set the basis for Luria’s neuropsychology and also for all of the other authors mentioned in this article, except for Brodmann (1909). Texts, articles, and books written before 1973 by Luria and his collaborators were used here, which may include modern data in newer editions.

For the post-Luria articles, the words searched in the titles of articles published after 1973 were “parietal lobe” AND “neuropsychology” using the advanced search in the CAPES database, which indexes articles publishers, including PubMed, Scopus, and Elsevier. Initially, 1,627 papers published from 1973 to 2023 were found. Articles on “alien hand syndrome,” “apraxia,” “attention and negligence,” “cytoarchitecture of the parietal lobes,” “dyscalculia,” “memory,” and “organization of the movement” were manually selected, while duplicates were excluded, with 119 papers remaining after this step. Among the papers considered to be most adequate for the goal of this work, priority was first given based upon how recently the article was published and then upon the quality of the journal in which the article was published (i.e., for articles with the same subject, the more recent articles were prioritized, and for equally recent articles with the same subject, those from best journal based upon impact factor were selected).

**Figure 1:**
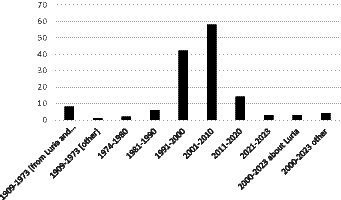
Number of selected papers plotted by publication year and in relation to Luria’s studies on neuropsychology and parietal lobes.

**Figure 2:**
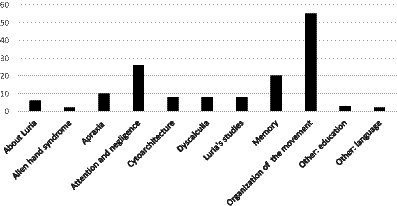
Number of the texts that contain the studied subjects in this work.

A group of papers from 2020 and later that link Luria with modern studies were collated separately under “2000–2023 about Luria,” and “1909–1973 [other]” includes articles on education and language that were used to justify some affirmations added to Brodmann’s (1909) article. Altogether, 138 texts were used in this work (Figures 1, 2).

## 3. Results

Most of the texts evaluated in this work were on parietal lobe and neuropsychology and utilized approaches such as image studies and lesions without necessary consideration to Luria’s studies. These were the first 130 s articles. Besides those, there were 8th specific texts from Luria’s studies (including his book and papers), and three modern studies from 1973 onwards that cited Luria. The articles hereby mentioned were published from 1909 to 2023, with periods of abundance and scarcity on parietal neuropsychology studies (Figure 1).

According to Figure 1, the number of articles produced increased from 1909 to 2010. However, after this time, the number of papers on the neuropsychology of the parietal lobe greatly decreased. Most of the articles about the neuropsychology of the parietal lobe were on the “organization of the movement” with 55 articles; 26 were on “attention and negligence”; 20 on “memory”; 10 were on “apraxia”; eight each on “dyscalculia,” “cytoarchitecture,” and “Luria’s studies”; and two were on “alien hand syndrome.” There were also six texts “about Luria” from the year 2000; three on “other: education” and two on “other: language” (Table 1; Figure 2). There was remarkable intersection among some of the subjects, in special between “attention and negligence” to “organization of the movement.”

**Table 1:** Studied texts in this review and their subjects.

	Texts	Subjects
1.	Aversi-Ferreira, T.A., Tamaishi-Watanabe, B.H., Magri, M.P.F., Aversi-Ferreira, R.A.G.M.F. (2019). Neuropsychology of the temporal lobe: Luria’s and contemporary conceptions. *Dement. Neuropsychol.* 13, 251–258. doi:10.1590/1980-57642018dn13-030001	About Luria
2.	Sugahara, C., Silveira, B. F., Azevedo, A. S. F., Macena, B. B., Aversi-Ferreira, T. A. (2021). The role of the second brain functional unit II on the memory’s process: A neuropsychological Luria’s perspective. *Research, Society and Development*. 10, e27010917957. doi: 10.33448/rsd-v10i9.17957	About Luria
3.	Cordeiro-de-Oliveira, K., Souza-Couto, D., Caixeta, M., Caixeta, V., Aversi-Ferreira, T.A. (2021). Neuropsychology of the frontal lobe and III functional brain unit: A Luria’s studies and perspectives for the clinic approach. *Research, Society and Development*. 10,1–10. doi: 10.33448/rsd-v10i7.16760	About Luria
4.	Hazin, I., and Falcão, J.T.R. (2014). Luria’s neuropsychology in the 21st century: Contributions, advancements, and challenges. *Psychology & Neuroscience*, 7, 433–434. doi: 10.3922/j.psns.2014.4.01	About Luria
5.	Lamdan, E., and Yasnitsky, A. (2013). Back to the future: toward Luria’s holistic cultural science of human brain and mind in a historical study of mental retardation. *Front. Hum. Neurosci*. 7, 1–3. doi: 10.3389/fnhum.2013.00509	About Luria
6.	Oliveira, M.K., and Rego, T.C. (2010). Aver Contribuições da perspectiva histórico-cultural de Luria para a pesquisa contemporânea. *Educação E Pesquisa*. 36, 107–121. doi: 10.1590/S1517-97022010000400009	About Luria
7.	Carrilho, P.E.M., Caramelli, P., Cardoso, F., Barbosa, E.R., Buchpiguel, C.A., Nitrini, R. (2001). Involuntary hand levitation associated with parietal damage. *Arq Neuropsiquiatr*. 59, 521–525. doi: 10.1590/s0004-282x2001000400007	Alien hand syndrome
8.	Barbieri, C., and De Renzi, E. (1988). The executive and ideational components of apraxia. Cortex. 24, 535–544. doi: 10.1016/s0010-9452(88)80047-9	Apraxia
9.	Fontana, A.P., Kilner, J.M., Rofrigues, E.C., Joffily, M., Nighoghossian, N., Vargas, C.D., Sirigu, A. (2012). Role of the parietal cortex in predicting incoming actions. *NeuroImage*. 59, 556–564. doi: 10.1016/j.neuroimage.2011.07.046	Apraxia
10.	Gibb, W.R.G., Luthert, P.J., Marsden, C.D. (1989). Corticobasal degeneration. *Brain*. 112, 1,171–1,192. doi: 10.1093/brain/112.5.1171	Apraxia
11.	Goldenberg, G. (2009). Apraxia and the parietal lobes. *Neuropsychologia*. 47, 1,449–1,459. doi: 10.1016/j.neuropsychologia.2008.07.014	Apraxia
12.	Makuuchi, M., Kaminaga, T., Sugishita, M. (2003). Both parietal lobes are involved in drawing: a functional MRI study and implications for constructional apraxia. *Brain Res Cogn Brain Res*. 16, 338–347. doi: 10.1016/s0926-6410(02)00302-6	Apraxia
13.	Merians, A.S., Clark, M., Poizner, H., Jacobs, D.H., Adair, J.C., Macauley, B., Rothi, L.J.G., Heilamn, K.M. (1995). Apraxia differs in corticobasal degeneration and left-parietal stroke: a case study. *Brain and Cognition*. 40, 314–335. doi: 10.1006/brcg.1999.1084.	Apraxia
14.	Niessen, E., Fink, G.R., Weiss, P.H. (2014). Apraxia, pantomime and the parietal cortex. *NeuroImage: Clinical*. 5, 42–52. doi: 10.1016/j.nicl.2014.05.017	Apraxia
15.	Poizner, H., Clark, M., Merians, A. S., Macauly, B., Rothi, L., Heilman, K. M. (1995). Joint coordination deficits in limb apraxia. *Brain.* 118. p. 227–242. doi: 10.1093/brain/118.1.227.	Apraxia
16.	Riley, D.E., Lang, A.E., Lewis, M.B., Resch, L., Ashby, P., Hornykiewicz, O., Black, S. (1990). Cortical-basal degeneration. *Archives of Neurology*. 40, 1,203–1,212. doi: 10.1212/wnl.40.8.1203	Apraxia
17.	Rinnie, J.O., Lee, M.S., Thompson, P.D., Marsden, C.D. (1994). Corticobasal degeneration: A clinical study of 36 cases. *Brain*. 117, 1,183–1,196. doi: 10.1093/brain/117.5.1183	Apraxia
18.	Clark, V.P., Fannos, S., Lai, S., Benson, R., Bauer, L. (2000). Responses to rare visual target and distractor stimuli using event-related fMRI. *J. Neurophysiol. 83*, 3,133–3,139. doi: 10.1152/jn.2000.83.5.3133	Attention and negligence
19.	Culham, J.C., and Valyear, K.F. (2006). Human parietal cortex in action. C*urr. Opin. Neurobiol.* 16, 205–212. doi: 10.1016/j.conb.2006.03.005	Attention and negligence
20.	Danckert, J., and Ferber, S. (2006). Revisiting unilateral neglect. *Neuropsychologia.* 44, 987–1,006. doi: 10.1016/j.neuropsychologia.2005.09.004	Attention and negligence
21.	Delis, D.C., Robertson, L.C., Efron, R. (1986). Hemispheric specialization of memory for visual hierarchical stimuli. *Neuropsychologia*. 24, 205–214. doi: 10.1016/0028-3932(86)90053-9	Attention and negligence
22.	Driver, J., and Mattingley, J.B. (1998). Parietal neglect and visual awareness. *Nature Neurosci.* 1, 17–22. doi: 10.1038/217	Attention and negligence
23.	Hilgetag, C.C., Théoret, H., Pascual-Leone, A. (2001). Enhanced visual spatial attention ipsilateral to rTMS-induced ‘virtual lesions’ of human parietal cortex. *Nature Neurosci*. 4, 953–957. doi: 10.1038/nn0901-953	Attention and negligence
24.	Linden, D.E., Prvulovic, D., Formisano, E., Vollinger, M., Zanella, F.E., Goebel, R., Dierks, T. (1999). The functional neuroanatomy of target detection: an fMRI study of visual and auditory oddball tasks. *Cereb. Cortex*. 9, 815–823. doi: 10.1093/cercor/9.8.815	Attention and negligence
25.	Malhotra, P., Coulthard, E., Husain, M. (2009). Role of right posterior parietal cortex in maintaining attention to spatial locations over time. *Brain.* 132, 645–660. doi: 10.1093/brain/awn350	Attention and negligence
26.	Marois, R., Leung, H.C., Gore, J.C. (2000b). A stimulus-driven approach to object identity and location processing in the human brain. *Neuron*. 25, 717–728. doi: 10.1016/s0896-6273(00)81073-9	Attention and negligence
27.	Mesulam, M.M. (1999). Spatial attention and neglect: parietal, frontal, and cingulate contributions to the mental representation and attentional targeting of salient extrapersonal events. *Philos Trans R Soc Lond B Biol Sci*. 354, 1,325–46. doi: 10.1098/rstb.1999.0482.	Attention and negligence
28.	Mevorach, C., Humphreys, G.W., Shalev, L. (2006a). Effects of saliency, not global dominance, in patients with left parietal damage. *Neuropsychologia*. 44, 307–319. doi: 10.1016/j.neuropsychologia.2005.04.015	Attention and negligence
29.	Mevorach, C., Humphreys, G.W., Shalev, L. (2006b). Opposite biases in salience-based selection for the left and right posterior parietal cortex. *Nature Neuroscience.* 9, 740–742. doi: 10.1038/nn1709	Attention and negligence
30.	Navon, D. (1997). Forest before trees: the precedence of global features in visual perception. *Cognitive Psychology.* 9, 353–383. doi: 10.1016/0010-0285(77)90012-3	Attention and negligence
31.	Pisella, L., Berberovic, N., Mattingley, J.B. (2004). Impaired working memory for location but not for colour or shape in visual neglect: a comparison of parietal and non-parietal lesions. *Cortex*. 40, 379–390. doi: 10.1016/s0010-9452(08)70132-1	Attention and negligence
32.	Posner, M.I., Walker, J.A., Friedrich, F.J., Rafal, R.D. (1984). Effects of parietal injury on covert orienting of attention. *J Neurosci*. v.4. p.1863–1874. doi: 10.1523/JNEUROSCI.04-07-01863.1984	Attention and negligence
33.	Rees, G., Wojciulik, E., Clarke, K., Husain, M., Frith, C., Driver, J. (2000). Unconscious activation of visual cortex in the damaged right hemisphere of a parietal patient with extinction. *Brain.* 123, 1,624–1,633. doi: 10.1093/brain/123.8.1624.	Attention and negligence
34.	Rossetti, Y., Rode, G., Pisella, L., Farne, A., Li, L., Boisson, D., Perenin, M.T. (1998). Prism adaptation to a rightward optical deviation rehabilitates left hemispatial neglect. *Nature.* 395, 166–169. doi: 10.1038/25988	Attention and negligence
35.	Striemer, C., Blangero, A., Rossetti, Y., Boisson, D., Rode, G., Salemme, R., Vighetto, A., Pisella, L., James, D. (2008). Bilateral parietal lesions disrupt the beneficial effects of prism adaptation: evidence from a patiente with optic ataxia. *Exp. Brain Res*. 187, 295–302. doi: 10.1007/s00221-008-1303-2	Attention and negligence
36.	Vecera, S.P., and Flevaris, A.V. (2005). Attentional control parameters following parietal-lobe damage: evidence from normal subjects. *Neuropsychologia.* 43, 1,189–1,203. doi: 10.1016/j.neuropsychologia.2004.10.009	Attention and negligence
37.	Buxbaum, L.J., Kyle, K., Grossman, M., Coslett, H.B. (2007). Left inferior parietal representations for skilled hand–object interactions: Evidence from stroke and corticobasal degeneration. *Cortex.* 43, 411–423. doi: 10.1016/s0010-9452(08)70466-0	Attention and negligence; organization of the movement
38.	Coull, J.T., and Frith, C.D. (1998). Differential activation of right superior parietal cortex and intraparietal sulcus by spatial and nonspatial attention. *Neuroimage*. 8, 176–187. doi: 10.1006/nimg.1998.0354	Attention and negligence; organization of the movement
39.	Coull, J.T., and Nobre, A.C. (1998). Where and when to pay attention: the neural systems for directing attention to spatial locations and to time intervals as revealed by both PET and fMRI. *J. Neurosci.* 18, 7,426–7,435. doi: 10.1523/JNEUROSCI.18-18-07426.1998	Attention and negligence; organization of the movement
40.	Downar, J., Crawley, A.P., Mikulis, D.J., Davis, K.D. (2002). A cortical network sensitive to stimulus salience in a neutral behavioral context across multiple sensory modalities*. J. Neurophysiol*. 87, 615–620. doi: 10.1152/jn.00636.2001	Attention and negligence; organization of the movement
41.	Husain, M., and Nachev, P. (2006). Space and the parietal cortex. *Trends in Cognitive Sciences*. 11, 30–36. doi: 10.1016/j.tics.2006.10.011	Attention and negligence; organization of the movement
42.	Marois, R., Chun, M.M, Gore, J.C. (2000a). Neural correlates of the attentional blink. *Neuron*. 28, 299–308. doi: 10.1016/s0896-6273(00)00104-5.	Attention and negligence; organization of the movement
43.	Wojciulik, E., and Kanwisher, N. (1999). The generality of parietal involvement in visual attention. *Neuron*. 23, 747–764. doi: 10.1016/s0896-6273(01)80033-7	Attention and negligence; organization of the movement
44.	Binkofski, F., Buccino, G., Dohle, C., Seitz, R.J., Freuns, H.J. (1999). Mirror agnosia and mirror ataxia constitute different parietal lobe disorders. *Annals of Neurology*. 46, 51–61. doi: 10.1002/1531-8249(199907)46:1 < 51::aid-ana9 > 3.0.co;2-q	Cytoarchitecture
45.	Brodmann, K. (1909). *Vergleichende Lokalisationslehre der Großhirnrinde.* Barth, Leipzig.	Cytoarchitecture
46.	Casper, S., Geyer, S., Schleicher, R.A., Mohlberg, H., Amunts, K., Zilles, K. (2006). The human inferior parietal cortex: cytoarchitectonic parcellation and interindividual variability. *NeuroImage*. 33, 430–448. doi: 10.1016/j.neuroimage.2006.06.054	Cytoarchitecture
47.	Nickel, J., and Seitz, R.J. (2005). Functional clusters in the human parietal cortex as revealed by an observer-independent meta-analysis of functional activation studies. *Anat Embryol.* 210, 463–472. doi: 10.1007/s00429-005-0037-1	Cytoarchitecture
48.	Wiesel T.N., and Hubel, D.H. (1963). Single-cell responses in striate cortex of kittens deprived of vision in one eye. *J Neurophysiol*. 26, 1,003–1,017. doi: 10.1152/jn.1963.26.6.1003	Cytoarchitecture
49.	Aversi-Ferreira, T.A., Araújo, M.F.P., Lopes, D. B., Nishijo, H. (2010). History, cytoarchitecture and neurophysiology of human and non human primates’ parietal loba – a review. *Dement. Neuropsychol.* 4, 173–180. doi: 10.1590/S1980-57642010DN40300005	Cytoarchitecture; about Luria
50.	Culham, J.C., and Kanwisher, N.G. (2001). Neuroimaging of cognitive functions in human parietal cortex. *Curr Opin Neurobiol*. 11, 157–163. doi: 10.1016/s0959-4388(00)00191-4	Cytoarchitecture; Organization of the movement
51.	Duhamel, J.R., Colby, C.L., Goldberg, M.E. (1992). The updating of the representation of visual space in parietal cortex by intended eye movements. *Science.* 3, 90–92. doi: 10.1126/science.1553535	Cytoarchitecture; Organization of the movement
52.	Ansari, D., Fugelsang, J.A., Dhital, B., Venkatraman, V. (2006). Dissociating response conflict from numerical magnitude processing in the brain: An event-related fMRI study. *Neuroimage*. 32, 799–805. doi: 10.1016/j.neuroimage.2006.04.184	Dyscalculia
53.	Cohen, L., Dehaene, S., Chochona, F., Lehericyc, S., Naccache, L. (2000). Language and calculation within the parietal lobe: a combined cognitive, anatomical and fMRI study. *Neuropsychologia*. 38, 1,426–1,440. doi: 10.1016/s0028-3932(00)00038-5	Dyscalculia
54	Dehaene, S., Piazza, M., Pinel, P., Cohen, L. (2003). Three parietal circuits for number processing. *Cog Neuropsychol*. 20, 487–506. doi: 10.1080/02643290244000239	Dyscalculia
55.	Kadosh, R.C., Kadosh, K.C., Schuhmann, T., Kaas, A., Goebel, R., Henik, A., Sack, A.T. (2007). Virtual dyscalculia induced by parietal-lobe TMS impairs automatic magnitude processing. *Current Biology*. 17, 689–693. doi: 10.1016/j.cub.2007.02.056	Dyscalculia
56.	Koontz, K.L., and Berch, D.B. (1996). Identifying simple numerical stimuli: Processing inefficiencies exhibited by arithmetic learning disabled children. *Math. Cogn*. 2, 1–23. doi: 10.1080/135467996387525	Dyscalculia
57.	Rivera, S.M., Reiss, A.L., Eckert, M.A., Menon, V. (2005). Developmental changes in mental arithmetic: evidence for increased functional specialization in the left inferior parietal córtex. *Cerebral Córtex*. 15, 1779–1790. doi: 10.1093/cercor/bhi055	Dyscalculia
58.	Rubinsten, O., and Henik, A. (2005). Automatic activation of internal magnitudes: A study of developmental dyscalculia. *Neuropsychology*. 19, 641–648. doi: 10.1037/0894-4105.19.5.641	Dyscalculia
59.	Simon, O., Mangin, J.F., Cohen, L., Bihan, D.L., Dehaene, S. (2002). Topographical layout of hand, eye, calculation, and language-related areas in the human parietal lobe. *Neuron*. 33, 475–487. doi: 10.1016/s0896-6273(02)00575-5	Dyscalculia; alien hand syndrome
60.	Luria, A.R., and Rapoport, M.Y. (1962). Regional symptoms of disturbance of the higher cortical function in intracerebral tumors of the left temporal lobe. *Vopr Neirokhir*. 4, 37–41.	Luria’s studies
61.	Luria, A.R., Simernitskaya, E.G., Tubylevich, B. (1970). The structure of psychological processes in relation to cerebral organization. *Neuropsychologia*. 8, 13–19. doi: 10.1016/0028-3932(70)90022-9	Luria’s studies
62.	Luria, A.R. (1996). *Higher cortical functions in man*. New York: Basic Books.	Luria’s studies
63.	Luria, A.R. (1971). Memory disturbances in local brain lesions. *Neuropsychologia*. 9, 367–375. doi: 10.1016/0028-3932(71)90001-7	Luria’s studies
64.	Luria, A.R. (1979). *The Making of Mind Cambridge*: Harvard University Press.	Luria’s studies
65.	Luria, A.R. (1987b). *The Man with a Shattered World: The History of a Brain Wound*. Cambridge: Harvard University Press.	Luria’s studies
66.	Luria, A.R. (1987a). *The Mind of a Mnemonist: A Little Book about a Vast Memory*. Cambridge: Harvard University Press.	Luria’s studies
67.	Luria, A.R. (1973). *The working brain: An introduction to neuropsychology*. London: Ed. Basic Books.	Luria’s studies
68.	Baddeley, A. (2000). The episodic buffer: A new component of working memory? *Trends in Cognitive Sciences*. 4, 417–423. doi: 10.1016/s1364-6613(00)01538-2	Memory
69.	Barch, D.M., Braver, T.S., Nystrom, L.E., Forman, S.D., Noll, D.C., Cohen, J.D. (1997). Dissociating working memory from task difficulty in human prefrontal cortex. *Neuropsychologia*. 35, 1,373–1,380. doi: 10.1016/s0028-3932(97)00072-9	Memory
70.	Berryhill, M.E., Chein, J., Olson, I.R. (2011). At the intersection of attention and memory: The mechanistic role of posterior parietal lobe in working memory. *Neuropsychologia*. 29, 1,306–1,315. doi: 10.1016/j.neuropsychologia.2011.02.033	Memory
71.	Berryhill, M.E., and Olson, I.R. (2008). Is the posterior parietal lobe involved in working memory retrieval? Evidence from patients with bilateral parietal lobe damage. *Neuropsychologia*. 46, 1775–1786. doi: 10.1016/j.neuropsychologia.20 08.03.005.	
72.	Brazdil, M., Mikl, M., Marecek, R., Krupa, P., Rektor, I. (2007). Effective connectivity in target stimulus processing: Adynamic causal modeling study of visual oddball task. *NeuroImage.* 35, 827–835. doi: 10.1016/j.neuroimage.2006. 12.020	Memory
73.	Cavanna, A.E., and Trimble, M.R. (2006). The precuneus: a review of its functional anatomy and behavioural correlates. Brain. 129, 564–583. doi: 10.1093/brain/awl004	Memory
74.	Ciaramelli, E., Faggi, G., Scarpazza, C., Mattioli, F., Spaniol, J., Ghetti, S., Mascovitch, M. (2017). Subjective recollection independent from multifeatural context retrieval following damage to the posterior parietal cortex. *Cortex*. 91, 114–125. doi: 10.1016/j.cortex.2017.03.015	Memory
75.	Davidson, P.S.R., Anaki, D., Ciaramelli, E., Cohn, M., Kim, A.S.N., Murphy, K.J., Troyer, A.K., Moscovitch, M., Levine, B. (2008). Does lateral parietal cortex support episodic memory? Evidence from focal lesion patients. *Neuropsychologia.* 46, 1743–1755, doi: 10.1016/j.neuropsychologia.2008.01.011	Memory
76.	Honey, G.D., Bullmore, E.T., Sharma, T. (2000). Prolonged reaction time to a verbal working memory task predicts increased power of posterior parietal cortical activation. *NeuroImage*. 12, 495–503. doi: 10.1006/nimg.2000.0624	Memory
77.	Jonides, J., Schumacher, E.H., Smith, E.E., Koeppe, R.A., Awh, E., Reuter-Lorenz, P.A., Marshuetz, C., Willis, C.R. (1998). The role of the parietal cortex in verbal working memory. *J. Neurosci*. 18, 5,026–5,034. doi: 10.1523/JNEUROSCI.18-13-05026.1998	Memory
78.	Marlene, B., and Geng, J.J. (2004). Parietal cortex and attention. *Curr Opin Neurobiol*. 14, 212–217. doi: 10.1016/j.conb.2004.03.012	Memory
79.	Olesen, P.J., Westerberg, H., Klingberg, T. (2004). Increased prefrontal and parietal activity after training of working memory. *Nature Neuroscience*. 7, 75–79. doi: 10.1038/nn1165	Memory
80.	Olson, I.R., and Berryhill, M. (2009). Some surprising findings on the involvement of the parietal lobe in human memory. *Neurobiol. Learn. Mem.* 91, 155–165. doi: 10.1016/j.nlm.2008.09.006	Memory
81.	Owen, A.M., McMillan, K.M., Laird, A.R., Bullmore, E. (2005). N-back working memory paradigm: a meta-analysis of normative functional neuroimaging studies. *Human Brain Mapping*. 25, 46–59. doi: 10.1002/hbm.20131	Memory
82.	Sandrini, M., Fertonani, A., Cohen, L.G., Miniussi, C. (2012). Double dissociation of working memory load effects induced by bilateral parietal modulation. *Neuropsychologia*. 30, 396–402. doi: 10.1016/j.neuropsychologia.2011.12.011	Memory
83.	Sesieri, C., Capotosto, P., Tosoni, A., Romani, G.L., Corbetta, M. (2013). Interference with episodic memory retrieval following transcranial stimulation of the inferior but not the superior parietal lobule. *Neuropsychologia*. 51, 900–906. doi: 10.1016/j.neuropsychologia.2013.01.023	Memory
84.	Skinner, E.I., and Fernandes, M.A. (2007). Neural correlates of recollection and familiarity: A review of neuroimaging and patient data. *Neuropsychologia.* 45, 2,163–2,179. doi: 10.1016/j.neuropsychologia.2007.03.007	Memory
85.	Todd, J.J., and Marois, R. (2004). Capacity limit of visual short-term memory in human posterior parietal cortex. *Nature*. 428, 751–754. doi: 10.1038/nature02466	Memory
86.	Vilberg, K.L., and Rugg, M.D. (2008). Memory retrieval and the parietal cortex: A review of evidence from a dual-process perspective. *Neuropsychologia*. 46, 1787–1799. doi: 10.1016/j.neuropsychologia.2008.01.004	Memory
87.	Wilding, E. L., and Rugg, M. D. (1996). An event-related potential study of recognition memory with and without retrieval of source. *Brain: A Journal of Neurology*. 119, 889–905. doi: 10.1093/brain/119.3.889	Memory
88.	Aflalo, T., Kellis, S., Klaes, C., Lee, B., Shi, Y., Pejsa, K., Shanfield, K., Hayes-Jackson, S., Aisen, M., Heck, C., Liu, C., Andersen, R.A. (2015). Decoding motor imagery from the posterior parietal cortex of a tetraplegic human. *Science*. 348, 906–910. doi: 10.1126/science.aaa5417	Organization of the movement
89.	Andersen, R.A., and Bueno, C. (2002). Intentional maps in posterior parietal cortex. Annu. Rev. Neurosci. 25, 189–220. doi: 10.1146/annurev.neuro.25.112701. 142,922	Organization of the movement
90.	Andersen, R.A., Kellis, S., Klaes, C., Aflalo, T. (2014). Toward more versatile and intuitive cortical brain-machine interfaces. *Curr. Biol.* 24, R885-R897. doi: 10.1016/j.cub.2014.07.068	Organization of the movement
91.	Blakemore, S.J., and Sirigu, A. (2003). Action prediction in the cerebellum and in the parietal lobe. *Ex Brain Res.* 153, 239–245. doi: 10.1007/s00221-003-1597-z	Organization of the movement
92.	Bonini, L., Rozzi, S., Serventi, F.U., Simone, L., Ferrari, P.F., Fogassi, L. (2010). Ventral premotor and inferior parietal cortices make distinct contribution to action organization and intention understanding. *Cerebral Cortex.* 20, 1,372–1,385. doi: 10.1093/cercor/bhp200	Organization of the movement
93.	Carota, F., Desmurget, M., Sirigu, A. (2010). “Forward modeling mediates motor awareness”, in Conscious Will and Responsibility, ed. W. Sinnott-Armstrong and L.Nadel (Oxford Univ. Press), 97–108.	Organization of the movement
94.	Corbetta, M., Kincade, M., Ollinger, J.M., Mcavoy, M.P., Shulman, G.L. (2000). Voluntary orienting is dissociated from target detection in posterior parietal cortex. *Nature Neurosci*. 3, 292–297. doi: 10.1038/73009	Organization of the movement
95.	Daprati, E., Sirigu, A., Nico, D. (2010). Body and movement: Consciousness in the parietal lobes. *Neuropsychologia*. 48, 756–762. doi: 10.1016/j.neuropsychologia.2009.10.008	Organization of the movement
96.	De Renzi, E., Faglioni, P., Lodesani, M., Vecci, A. (1983). Performance of left brain-damaged patients on imitation of single movements and motor sequences. Frontal and parietal-injured patients compared. *Cortex.* 19, 333–343. doi: 10.1016/s0010-9452(83)80004-5	Organization of the movement
97.	Desmurget, M., and Sirigu, A. (2012). Conscious motor intention emerges in the inferior parietal lobule. *Curr Opin Neurobiol*. 22, 1,004–1,011. doi: 10.1016/j.conb.2012.06.006	Organization of the movement
98.	Desmurget, M., Reilly, K.T., Richard, N., Szathmari, A., Sirigu, A. (2009). Movement intention after parietal cortex stimulation in humans. *Science.* 324, 811–813. doi: 10.1126/science.1169896	Organization of the movement
99.	Desmurget, M., and Sirigu, A. (2009). A parietal-premotor network for movement intention and motor awareness. *Trends in Cognitive Sciences.* 13, 411–419. doi: 10.1016/j.tics.2009.08.001	Organization of the movement
100.	Downar, J., Crawley, A.P., Mikulis, D.J., Davis, K.D. (2001). The effect of task relevance on the cortical response to changes in visual and auditory stimuli: An event-related fMRI study. *Neuroimage*. 14, 1,256–1,267. doi: 10.1006/nimg.2001.0946	Organization of the movement
101.	Fogassi, L., and Luppino, G. (2005). Motor function of the parietal lobe. *Current Opinion in Neurobiology.* 15, 626–631. doi: 10.1016/j.conb.2005.10.015	Organization of the movement
102.	Fridman, E.A., Desmurget, M., Sirigu, A. (2011). “From conscious motor intention to movement awareness”, in *Characterizing Consciousness: From Cognition to the Clinic?*, ed. S. Dehaene and Y. Christen (Fondation IPSEN, Springer), p. 191–198.	Organization of the movement
103.	Geschwind, N (1975). The apraxias: Neural mechanisms of disorders of learned movements. *American Scientist.* 63, 188–195.	Organization of the movement
104.	Giesbrecht, B., Woldorff, M.G., Song, A.W., Mangun, G.R. (2003). Neural mechanisms of top-down control during spatial and feature attention. *Neuroimage.* 19, 496–512. doi: 10.1016/s1053-8119(03)00162-9	Organization of the movement
105.	Goodale, M.A., and Milner, A.D. (1992). Separate visual pathways for perception and action. *Trends in Neuroscience*. 15, 20–25. doi: 10.1016/0166-2236(92)90344-8	Organization of the movement
106.	Haggard, P. (2005). Conscious intention and motor cognition. *Trends Cogn Sci*. 9, 290–295. doi: 10.1016/j.tics.2005.04.012	Organization of the movement
107.	Haggard, P. (2009) Neuroscience. The sources of human volition. *Science.* 324, 731–733. doi: 10.1126/science.1173827	Organization of the movement
108.	Haggard, P., and Eimer, M. (1999). On the relation between brain potentials and the awareness of voluntary movements. *Exp. Brain Res.* 126, 128–133. doi: 10.1007/s002210050722.	Organization of the movement
109.	Hauschild, M., Mulliken, G. H., Fineman, I., Loeb, G.E., Andersen, R. A. (2012). Cognitive signals for brain-machine interfaces in posterior parietal cortex include continuous 3D trajectory commands. *Proc. Natl. Acad. Sci*. 109, 17,075–17,080. doi: 10.1073/pnas.1215092109	Organization of the movement
110.	Heilman, K.M., Rothie, L.J., Valenstein, E. (1982). Two forms of ideomotor apraxia. *Neurology*. 32, 342–346. doi: 10.1212/wnl.32.4.342	Organization of the movement
111.	Husain, M., Stein, J. (1988). Rezsö Bálint and his most celebrated case. *Archives of Neurology*. 45, 89–93. doi: 10.1001/archneur.1988.00520250095029	Organization of the movement
112.	Hwang, E.J., Hauscild, M., Wilke, M., Andersen, R.A. (2012). Inactivation of the parietal reach region causes optic ataxia, impairing reaches but not saccades. *Neuron*. 76, 1,021–1,029. doi: 10.1016/j.neuron.2012.10.030	Organization of the movement
113.	Jeannerod, M., Arbib, M.A., Rizzolatti, G., Sakata, H. (1995). Grasping objects: the cortical mechanisms of visuomotor transformation. *Trends Neurosci.* 18, 314–320. doi: 10.1016/0166-2236(95)93921-J.	Organization of the movement
114.	Karnath, H.O., and Perenin, M.T. (2005). Cortical control of visually guided reaching: evidence from patients with optic ataxia. *Cerebral Cortex*. 15, 1,561–1,569. doi: 10.1093/cercor/bhi034	Organization of the movement
115.	Liu, T., Slotnick, S.D., Serences, J.T., Yantis, S. (2003). Cortical mechanisms of feature-based attentional control. *Cereb Cortex*. 13, 1,334–1,343. doi: 10.1093/cercor/bhg080	Organization of the movement
116.	Mulliken, G.H., Musallam, S., Andersen, R.A. (2008). Decoding trajectories from posterior parietal cortex ensembles. *J. Neurosci.* 28, 12,913–12,926. doi: 10.1523/JNEUROSCI.1463-08.2008	Organization of the movement
117.	Musallam, S., Corneil, B.D., Greger, B., Scherberger, H., Andersen, R. A. (2004). Cognitive control signals for neural prosthetics. *Science*. 305, 258–262. doi: 10.1126/science.1097938	Organization of the movement
118.	Pellijeff, A., Bonilha, L., Morgan, P.S., Mckenzie, K., Jackson, S.R. (2006). Parietal updating of limb posture: an event-related fMRI study. *Neuropsychologia*. 44, 2,685–2,690. doi: 10.1016/j.neuropsychologia.2006.01.009	Organization of the movement
119.	Rizzolatti, G., Fogassi, L., Gallese, V. (1997). Parietal cortex: From sight to action. *Current Opinion in Neurobiology*. 7, 562–567. doi: 10.1016/S0959-4388(97)80037-2	Organization of the movement
120.	Rothi, L.J.G., Ochipa, C., Heilman, K.M. (1991). A cognitive neurpsychological model of limb praxis. *Cognitive Neuropsychology*. 8, 443–458. doi: 10.1080/02643299108253382	Organization of the movement
121.	Rushworth, M.F.S., Nixon, P.D., Renowden, S., Wade, D.T., Passingham, R.E. (1997). The left parietal cortex and motor attention*. Neuropsychologia*. 35, 1,261–1,273. doi: 10.1016/s0028-3932(97)00050-x	Organization of the movement
122.	Sakata, H., Taira, M., Kusunoki, M., Murata, A., Tanaka, Y. (1997). The parietal association cortex in depth perception and visual control of hand action. *Trends Neurosci.* 20, 350–357. doi: 10.1016/s0166-2236(97)01067-9	Organization of the movement
123.	Sirigu, A., Cohen, L., Duhamel, J.R., Pillon, B., Dubois, B., Agid, Y. (1995). A selective impairment of hand posture for object utilization in apraxia. *Cortex*. 31, 41–55. doi: 10.1016/s0010-9452(13)80104-9	Organization of the movement
124.	Sirigu, A., Daprati, E., Ciancia, S., Giraux, P., Nighoghossian, N., Posada, A., Haggard, P. (2004). Altered awareness of voluntary action after damage to the parietal cortex. *Nature Neuroscience*. 7, 80–84. doi: 10.1038/nn1160	Organization of the movement
125.	Sirigu, A., Daprati, E., Pradat-Diehl, P., Franck, N., Jeannerod, M. (1999). Perception of self-generated movement following left parietal lesion. *Brain.* 122, 1867–1874. doi: 10.1093/brain/122.10.1867	Organization of the movement
126.	Snyder, L.H. (2000). Coordinate transformations for eye and arm. *Current Opinion in Neurobiology*. 10, 747–754. doi: 10.1016/S0959-4388(00)00152-5	Organization of the movement
127.	Tanaka, K. (1996). Inferotemporal cortex and object vision. *Annu Rev Neurosci.* 19, 109–139. doi: 10.1146/annurev.ne.19.030196.000545	Organization of the movement
128.	Vallar, G. (2007). Spatial neglect, Balint-Homes’ and Gerstmann’s syndrome, and other spatial disorders. *CNS Spectrums*. 12, 527–536. doi: 10.1017/s1092852900021271	Organization of the movement
129.	Vesia, M., and Crawford, D. (2012). Specialization of reach function in human posterior parietal cortex. *Exp. Brain. Res*. 221, 1–18. doi: 10.1007/s00221-012-3158-9	Organization of the movement
130.	Wolpert, D.M., Goodbody, S.J., Husain, M. (1998). Maintaining internal representations: The role of the human superior parietal lobe. *Nature Neuroscience*. 1, 529–533. doi: 10.1038/2245	Organization of the movement
131.	Yantis, S., Schwarzbach, J., Serences, J.T., Carlson, R.L., Steinmetz, M.A., Pekar, J.J., Courtney, S.M. (2002). Transient neural activity in human parietal cortex during spatial attention shifts. *Nat Neurosci.* 5, 995–1,002. doi: 10.1038/nn921	Organization of the movement
132.	Yantis, S., and Serences, J.T. (2003). Cortical mechanisms of space-based and object-based attentional control. *Curr Opin Neurobiol.* 13, 187–193. doi: 10.1016/s0959-4388(03)00033-3	Organization of the movement
133.	Behrmann, M., Geng, J.J., Shomstein, S. (2004). Parietal cortex and attention. *Curr Opin Neurobiol.* 14, 212–217. doi: 10.1016/j.conb.2004.03.012	Organization of the movement
134.	Alves, P. A., and Aversi-Ferreira, T. A. (2019). Comments on the problems solving methodology in education of civil engineering in Brazil. *R. Bras. Ens. Ci. e Tecnol*. 12, 134–153. doi: 10.3895/rbect.v12n1.7946.	Other: education
135.	Aversi-Ferreira, T., and Dias-Vieira, M. (2021). Comments about gifted students and their adjustment to the scholar-brazilian model. *Conjecturas.* 21, 532–543. doi: 10.53660/CONJ-406-217	Other: education
136.	Bastos, L.S., and Alves, M.P. (2013). As influências de Vygotsky e Luria à neurociência contemporânea e à compreensão do processo de aprendizagem. *Práxis*. 5, 41–53. doi: 10.25119/praxis-5-10-580	Other: education
137.	Aversi-Ferreira, T.A., Borges, K.C.M., Gonçalves-Mendes, M.T., Caixeta, L.F. (2021). Gross anatomy of the longitudinal fascicle of Sapajus sp. *PLoS ONE*. 16:e0252178. doi: 10.1371/journal.pone.0252178	Other: language
138.	Borges, K.C., Nishijo, H., Aversi-Ferreira, T.A., Ferreira, J.R., Caixeta, L.F. (2015). Anatomical Study of Intrahemispheric Association Fibers in the Brains of Capuchin Monkeys (Sapajus sp.). *Biomed Res Int.* 2015:648128. doi: 10.1155/2015/648128.	Other: language

## 4. Discussion

Due to the relevance of Luria’s studies and the discoveries of Russian psychology, a comparative analysis between modern data and the parietal lobe’s functions studied by Luria indicates the path of modern neuropsychology from the primordia of this science. Part of Vygotsky’s and Luria’s ideas and discoveries are used as the basis of educational psychology, mainly the socio-historical-cultural theory, to consider the influence of the environment on brain development and behavior.

In this way, some authors performed a comparison of Luria’s work and modern neuropsychology for the temporal, frontal, and parietal lobes (Aversi-Ferreira et al., 2010, 2019; Cordeiro-de-Oliveira et al., 2021; Sugahara et al., 2021), for education and neuroscience in general (Bastos and Alves, 2013; Lamdan and Yasnitsky, 2013; Hazin and Falcão, 2014; Alves and Aversi-Ferreira, 2019; Aversi-Ferreira et al., 2019; Aversi-Ferreira and Dias-Vieira, 2021). Aversi-Ferreira et al. (2010) focused on the cytoarchitecture of the parietal lobe, while this study takes a more neuropsychologic approach. Luria (1973) studied the brain structure to explain human and animal behavior, sometimes comparatively. If the brain is the foundation of the mind, then studying the cortex, especially the neocortex cytoarchitecture, is essential to understanding human and animal behavior (Aversi-ferreira et al., 2010).

### 4.1. Cytoarchitecture of parietal lobe

The human brain is very complex and difficult to understand. It has many connections and fibers that form a three-dimensional network, which could be the most complex structure in the universe. However, some imaging techniques can help researchers study the large bundles of fibers that connect different brain regions. For example, one of these bundles is the arcuate fascicle, which connects the Broca’s and Wernicke’s areas, involved in the production and comprehension of language (Borges et al., 2015; Aversi-Ferreira et al., 2021). On a smaller scale, the brain cortex is divided into layers that show how neurons are organized, but it is very difficult to trace the fibers that run through them.

The division of the brain into lobes does not reflect the complete physiology of its functions but only indicates the location of some basic functions, such as motor and sensory areas. However, complex functions cannot be strictly localized. In this sense, the second functional unit of the brain, according to Luria’s physiological division of the brain, is located in an area that includes the occipital, temporal, and, mainly, the parietal cortex in the lateral aspect. Higher activities in the brain are associated with non-specific functional neurons (i.e., non-modal ones; Luria, 1973).

The human parietal cortex is a granular heterotypic isocortex/neocortex, a highly differentiated structure with defined subareas and many connections (Culham and Kanwisher, 2001; Nickel and Seitz, 2005; Casper et al., 2006). Its inferior part integrates the somatosensory, visual, and auditory modalities and some higher cognitive functions (Casper et al., 2006; Aversi-Ferreira et al., 2010). The architecture of the cortices indicates the functional work in the brain and was used as one of the bases for determining the functional units proposed by Luria (1973), with the parietal cortex being the main structure of the second functional unit.

According to Luria’s proposal, the second functional unit of the brain has the function of receiving, analyzing, and storing information. It is located in the lateral part of the parietal neocortex and the neighboring temporal and occipital areas. In particular, it is responsible for obtaining and processing information from the body periphery that is modally specific (e.g., touch, pain, and temperature) that arrives in Brodmann’s areas 1, 2, and 3 (Brodmann, 1909). Similarly, part of Brodmann’s areas 41 and 42 receive auditory and vestibular information, and area 17 receives visual data located in the occipital cortex. These regions are structured by a primary projection area with a prominent layer IV rich in granular neurons that are modally specific (Wiesel and Hubel, 1963) and work to receive information and send it to other cortical areas, mainly the neighboring ones that are secondary areas in cytoarchitectural organization and are also modally specific.

The secondary modal parts are Brodmann’s areas 5 and 7; 21 and 22; 18 and part of 19, located in the parietal, temporal, and occipital lobes, respectively. The secondary cortices have very well-developed layers II and IV with many granular neurons and small pyramidal neurons in a similarly developed layer III, presenting predominantly associative features to perform synthetic functions.

According to Luria (1973), the second functional unit works under three laws: (1) a hierarchical structure in the cortical zones that depends on the primary zones and sends information to other secondary and tertiary areas in a progressive functional relationship to reach complex cognitive interpretation work; (2) a decrease in the specificity of the cortical zones (i.e., the primary zone is highly specific and decreases in specificity towards the secondary and finally the tertiary, which is a multimodal zone); and (3) a progressive lateralization of the functions (i.e., the information arrives at the primary zone, goes to the secondary, and then reaches the tertiary zones). As the tertiary zones are different in both hemispheres, some functions are more processed in one than in the other, generating concepts about the dominant hemisphere.

The posterior inferior part of the human parietal lobe is very complex with a multimodal structure, which is an association area that generates simultaneous synthesis unlike the modal structure of the occipital and temporal lobes (Luria, 1973). Therefore, the parietal lobe’s features pose challenges for interpreting neuroimaging of the physiology of this area (Culham and Kanwisher, 2001). Thus, logically, the analysis of lesions seems to be the best way to study this brain region.

In the non-modal area of the parietal lobe, lesions do not cause specific modal disturbances (Luria, 1973, 1987b). However, apraxia, agnosia, and Wernicke’s aphasia, which are derived from the abnormalities of the higher functions of the cortex, are the main problems resulting from parietal lesions. However, new types of agnosia and apraxia, such as mirror agnosia and ataxia caused by lesions near the postcentral sulcus and posterior parietal area, were discovered from lesion studies in this area (Binkofski et al., 1999).

Both disturbances are related to problems of visual-motor coordination and spatial representation (Duhamel et al., 1992). Luria described problems somewhat similar to those mentioned above in the lesions of the right parietal lobe, such as visual relations with the external environment.

### 4.2. Apraxia

Luria observed that lesions in different parts of the cortex could produce similar impairments of cognitive functions. However, he argued that with a thorough and careful analysis, it is possible to identify the specific location of the lesion that caused the dysfunction. One example of this situation is the case of kinetic and asymmetric apraxia, which results from the degeneration of the corticobasal region. This condition is characterized by muscle rigidity, involuntary movements, and a loss of cortical sensation (Gibb et al., 1989; Riley et al., 1990; Rinnie et al., 1994).

The apraxia observed in corticobasal degeneration is similar to that which is caused by a left parietal lobe lesion due to vascular accidents, such as ischemia or hemorrhage (Merians et al., 1995). Merians et al. (1995) compared a patient with corticobasal degeneration and a patient with a vascular lesion of the left parietal lobe, and he found significant differences between them. The former patient demonstrated a better articulation of movement in the spatial plane when instructed and without using a tool, while the latter patient showed more severe apraxic movements.

Both patients were apraxic when they performed movements with tools (Merians et al., 1995). The researchers concluded that corticobasal degeneration affects the whole cortex, while the vascular lesion was confined to the left parietal lobe, which according to Luria is responsible for spatial orientation (Luria, 1973). When spatial orientation is impaired, it results in a lack of movement organization in the spatial plane (Luria, 1973; Poizner et al., 1995).

Apraxia is characterized by errors in imitating gestures, difficulty in performing meaningful gestures on command, and failures in using tools or objects (Barbieri and De Renzi, 1988). Goldenberg (2009) showed that a lesion of the parietal lobe affects the imitation of meaningless gestures and the use of tools and is correlated with a loss of spatial relations between body parts and between the body and tools, rather than with problems in motor action as previously assumed (Goldenberg, 2009). However, recent studies suggest that imitation errors are associated with an anticipatory process that involves learning and predictive mechanisms, not just mirroring (Fontana et al., 2012).

Goldenberg’s theory confirms an observation of Luria that he made when studying patients with lesions of the left parieto-occipital lobe. Specifically, he identified constructive apraxia characterized by a lack of spatial orientation, leading to symptoms such as disorientation and difficulty in writing and recognizing objects. In addition to constructive apraxia, difficulty in performing gestures on command and memory loss were also identified, which together were deemed Gerstmann’s syndrome (Luria, 1973).

Constructional apraxia can be identified by disturbances in drawing performance. Drawing is thought to require the use of both parietal lobes, but individuals may differ in the degree to which they rely on each lobe (Luria, 1973). Studies carried out by Makuuchi et al. using functional magnetic resonance imaging (fMRI) identified that both parietal lobes are activated during both drawing and naming objects seen in a picture, and activation is more prominent left parietal lobe for most individuals (Makuuchi et al., 2003).

Pantomime is a classic examination procedure for identifying apraxia (Barbieri and De Renzi, 1988). Studies relate a deficit in handling tools to lesions of the left parietal lobe (Goldenberg, 2009), demonstrating that it stores motor schemes and activates them. When the inactivation is inadequate, pantomiming deficits arise, in which the object and its function partially lose their meaning for the individual, making imagined schemes and executed schemes difficult both with and without the tool (Niessen et al., 2014). This process was studied by Luria (1973).

### 4.3. Dyscalculia

Dyscalculia is characterized by difficulty in accessing numerical magnitudes, which can be triggered by brain lesions (Koontz and Berch, 1996; Rubinsten and Henik, 2005). Studies have determined the intraparietal sulcus as the central area for numerical processing (Dehaene et al., 2003; Ansari et al., 2006). Based on this information, Kadosh et al. (2007) used a technique called neuronavigation to temporarily disrupt this part of the brain in healthy people. The study found that this disruption caused dyscalculia-like symptoms, showing that this part of the brain is involved in automatic number processing (Kadosh et al., 2007). Other studies have shown that brain damage to another part of the brain called the left perisylvian region can lead to problems with calculating, reading, and speaking (Cohen et al., 2000). The studies have also shown that tasks that require both language and calculation skills activate this part of the brain as well as the left intraparietal sulcus (Simon et al., 2002).

Cohen et al. (2000) observed a bilateral activation of the parietal region and adjacent areas, suggesting that the right parietal lobe plays a role in arithmetic activities. In a topographic study conducted by Simon et al. (2002), activation was detected in the left intraparietal sulcus of the upper part of the posterior segment of the post-central sulcus, but in the right hemisphere, activation occurred in the horizontal segment of the intraparietal sulcus, which suggests a possible common network between language and arithmetic processes between the left and right parietal lobes. These results support Luria’s theory that math skills are not just located on one side of the brain but also depend on the other side of the brain for spatial and dimensional abilities (Luria, 1973).

The process of mental arithmetic activities involves different regions in the brains of children, adolescents, and adults. In adults, math skills are associated with the left parietal cortex, along with the supramarginal gyrus and adjacent anterior intraparietal sulcus. In younger people, activation occurs in the dorsal and ventrolateral prefrontal cortices, anterior cingulate gyrus, hippocampus, and dorsal basal ganglia, suggesting a greater use of working memory, attention, and declarative and procedural memory. This means that as people age, arithmetic processes become more focused on one part of the brain, the lower left parietal cortex (Rivera et al., 2005), which corroborates the studies conducted by Luria (1973) in which he demonstrates the association between left parietal lesions and mathematical dysfunctions in adults.

To understand the mathematical function performed by the left parietal cortex, one must consider the coexistence of three circuits in the parietal lobe, each associated with different arithmetic skills. Specifically, there are is a bilateral intraparietal circuit associated with a quantitative system; another circuit in the region in the left angular gyrus associated with verbal processing of numbers; and a circuit in the posterior superior parietal portion associated with spatial and non-spatial attention (Dehaene et al., 2003).

### 4.4. Alien hand syndrome

A study conducted by Carrilho et al. (2001) involved four patients with alien hand syndrome, a rare neurological disorder in which one hand acts involuntarily without the patient noticing. The symptoms include involuntarily grabbing and squeezing, touching one’s face or tearing one’s clothes, stuffing one’s mouth with food, preventing the unaffected hand from performing simple tasks, and poking and choking oneself. The results revealed extensive damage to the contralateral parietal cortex in the involved patients, reinforcing the theory that lesions in the parietal lobe may play a role in the genesis of involuntary hand levitation (Carrilho et al., 2001).

FMRI studies have also revealed a symmetrical activation of the parietal lobes in visuospatial tasks (grabbing, pointing, looking, and attention) using the right hand (Simon et al., 2002). According to Luria, the parietal region is responsible for complex syntheses and is related to spatial orientation, but in his reports no association was made between motor disorders and parietal lesions (Luria, 1973). However motor disorders may be related to difficulties in spatial orientation, causing disordered and apraxic movements.

### 4.5. Attention and negligence

After several studies, Buxbaum et al. found that the left inferior parietal lobe (IPL) mediates object representations in distinct ways. In the ventral stream, the left IPL is responsible for the representation of the object’s identity, while it mediates the spatial representations of the body in the dorsal pathway (Buxbaum et al., 2007). Another study conducted by Husain and Nachev showed that the IPL is also associated with the detection of salient events in repetitive flow (Linden et al., 1999; Clark et al., 2000; Marois et al., 2000b; Downar et al., 2002) and controls the attentional level over time, maintaining selective attention (Coull and Frith, 1998; Coull and Nobre, 1998; Wojciulik and Kanwisher, 1999; Marois et al., 2000b; Vogel and Machizawa, 2004) and participating in the ventral frontoparietal circuit together with the temporoparietal junction and intraparietal sulcus (Husain and Nachev, 2006).

Studies on the parameters that affect attention control in patients with lesions in the parietal areas and on healthy individuals in which a state similar to neglect, a condition in which patients ignore or fail to respond to stimuli on the opposite side of the lesion, was inducted showed deficits in “attentional capture” (Vecera and Flevaris, 2005). Related to this, Luria showed that brain lesions may lead to attention deficits (Luria, 1973).

In a similar study, Hilgetag et al. (2001) found phenomena commonly observed in patients with negligence, with a significant improvement in targets located ipsilaterally to the lesion (Hilgetag et al., 2001). One of the types of negligence is unilateral spatial agnosia, which was reported by Luria (1973). Spatial agnosia is characterized by a failure of the individual to explore the side opposite the lesion and react to stimuli that come from that side (Posner et al., 1984; Hilgetag et al., 2001), which is caused by damage to the right hemisphere (Mesulam, 1999). Previous studies have reported the involvement of a damaged parietal lobe in attention deficit when the target is located on the opposite side of the lesion, which indicates a connection to the parietal lobe in the process of selective attention (Posner et al., 1984). Studies reported by Malhotra et al. (2009) showed that patients with neglect whose lesions reached the right posterior parietal cortex (PPC) had attention deficits, evidencing the participation of the right parietal cortex in attending to spatial locations.

Luria (1973) reported that brain damage affects attentional process can modify the intensity of which a stimulus is perceived, where weak stimuli can be evaluated as strong stimuli. He also cited unilateral spatial agnosia as a consequence of lesions of the right parietal cortex, characterized by an unawareness of the left half of the visual field and even of one’s own body.

It is known that the cerebral hemispheres have different functions. While the right hemisphere is more involved in the processing of global visual information (Navon, 1997), the left hemisphere is more involved in processing local information (Delis et al., 1986). Moreover, the left parietal lobe is known to shift attention between local and global levels (Mevorach et al., 2006a).

An experiment carried out by Mevorach et al. (2006a) investigated the hypothesis that the left parietal lobe is associated with attentional control over hierarchical visual processing and is able to ignore irrelevant aspects of a stimulus when the target attribute is more prominent. Mevorach et al. tested patients with left parietal lobe lesions. However, the patients did not have difficulties in basic global or local identification but rather difficulties in modifying the hierarchy before the task. It was not possible to choose between global or local attention, showing that the left parietal lobe is fundamental for the network of selection and the monitoring of responses (Mevorach et al., 2006a). This observation was not highlighted in the studies by Luria (1973), who associated the functions of logical and mathematical reasoning with the left parietal lobe, not attributing the function of attention control and modulation of attentional systems to the parietal cortex (Luria, 1973).

Mevorach et al. (2006b) conducted another study that showed that the left and right posterior parietal cortices have different functions depending on the intensity of a stimulus. The right posterior parietal cortex (RPPC) handles the most intense stimuli, while the left posterior parietal cortex (LPPC) ignores them. This means that an irrelevant stimulus is first processed by the RPPC and then by the LPPC, which prevents the selection of irrelevant information. This is different from what has been seen in other studies, where the right posterior parietal cortex is also influenced by intense local stimuli and the left ignores intense global stimuli. This shows that the preference for global and local forms in the posterior parietal cortex can change depending on how intense the stimulus is (Mevorach et al., 2006b).

Visual attention is the ability to selectively process only some of the information that is present in the image that the retina captures (Wojciulik and Kanwisher, 1999). Visual neglect in patients with brain injuries is not caused by damage to the primary visual cortex but by damage to the opposite regions of the parietal lobe, especially the right IPL (Driver and Mattingley, 1998; Rees et al., 2000). In fact, a study on patients with lesions in the right IPL showed that both the left and right primary visual cortices were activated (Rees et al., 2000). However, these patients still had visual neglect, which shows that the inferior parietal cortex is essential for image processing (Driver and Mattingley, 1998; Rees et al., 2000). This information adds to the studies done by Luria (1973), who also attributed the function of attention and visual processing to the right parietal lobe.

The right parietal cortex is responsible for processing somatosensory information, especially visual information (Navon, 1997), while damage to this region can lead to neglect (Danckert and Ferber, 2006). Neglect also impairs working memory, affecting the ability to update and maintain spatial representations and to re-map spaces after eye movements (Pisella et al., 2004). Prism adaptation is a potential intervention for neglect that involves wearing prism glasses that shift the visual field (Rossetti et al., 1998). However, the underlying mechanism of prism adaptation is unclear. It has been suggested that the mechanism involves modifying the dysfunctional attentional orientation mediated by the superior parietal cortex, which is crucial for attention and eye and limb movements and has cerebellar connections that are essential for prism adaptation. Lesions to the superior parietal cortex prevent improvement in neglect after prism adaptation (Striemer et al., 2008).

### 4.6. Memory

Functional neuroimaging studies have shown that parietal regions are activated during episodic memory, particularly when an antecedent memory already exists (Wilding and Rugg, 1996). The lateral regions of the superior and inferior parietal lobes are crucial in memory retrieval (Vilberg and Rugg, 2008). The superior region reflects the relevance of old and new recognition tasks, while the inferior region is related to the recovery of successful memories, forming an “episodic buffer” (Baddeley, 2000; Brazdil et al., 2007).

The medial region of the parietal lobe plays a critical role in episodic memory. However, recent evidence demonstrates that the lateral posterior parietal cortex also has a significant role (Skinner and Fernandes, 2007). The angular gyrus in the IPL is activated during the recruitment of relevant information on physical memory and the primary care network, while the superior parietal lobe is activated during the recruitment of conceptual memories (Sesieri et al., 2013). Davidson et al. (2008) evaluated this hypothesis by examining data from patients with focal damage to their lateral posterior parietal cortex. The individuals had disordered conscious recall, which demonstrates the importance of this region for the recovery of episodic and working memories. Another study examined patients with lesions in the posterior parietal cortex, demonstrating the contribution of this area to the subjective experience and metamnemonic evaluation of memory content. Injured patients had problems recruiting multifactorial memory-reminders (Cavanna and Trimble, 2006; Ciaramelli et al., 2017).

Electrostimulation and neuroimaging were also used to study working memory. The researchers found that the more demanding the task, the greater the activation of the dominant side posterior parietal cortex. This activation cannot be attributed to an increase in difficulty, since it did not change sides. Therefore, the researchers hypothesized that different activation strategies are used, with the easiest tasks relying on a familiarity-based activation strategy and the more complex tasks requiring an updated strategy based on memory (Sandrini et al., 2012).

Confirming the participation of the parietal cortex in working memory, researchers used training to increase plasticity in the neural systems that underlie working memory. The results of fMRI showed increased activity in the middle frontal gyrus as well as in the superior and inferior parietal cortices (Olesen et al., 2004). Additionally, the activation of the posterior parietal cortex, the posterior region of the posterior temporal gyrus, and other areas of the brain were observed under conditions of storage and retrieval of verbal speech (Jonides et al., 1998).

Studies carried out through meta-analysis have shown that the activation of different cortical areas occurs during the recruitment of working memory. Evidence of marked activation was found in the lateral premotor cortex, dorsal cingulate cortex, medial premotor cortex, dorsolateral and ventrolateral prefrontal cortices, frontal poles, and medial and lateral posterior parietal cortices (Owen et al., 2005). The information obtained on the relationship between working memory and the parietal cortex contrasts with Luria’s work (Luria, 1973), in which working memory is not portrayed as one of the functions of this cortex. However, mnemonic dysfunctions associated with lesions of the left parieto-occipital cortex are associated with the loss of semantic integrity.

The posterior parietal cortex is a brain region that is involved in a wide variety of tasks involving short-term memory. Damage to this area can lead to memory deficits (Berryhill and Olson, 2008; Olson and Berryhill, 2009). A study by Todd and Marois (2004) found that PPC activity was greater when participants were asked to remember a visual stimulus than when they were asked to remember a non-visual stimulus. This suggests that the PPC may be specifically involved in the storage and maintenance of visual information in short-term memory, making it a key location for the mental representation of the visual world. Further studies by Berryhill and Olson (2008) and Berryhill et al. (2010) have confirmed the role of the PPC in attention-mediated mechanisms of short-term memory. These studies suggest that the PPC is a critical region for short-term memory, particularly for visual information.

While the role of the posterior parietal cortex in working memory is not fully understood. Some studies have suggested that the PPC helps to keep or shift attention to items and objects in working memory (Behrmann et al., 2004; Berryhill et al., 2011). However, patients with bilateral PPC lesions may have impaired working memory performance or rely on long-term memory instead (Berryhill et al., 2011). Moreover, the PPC activation during working memory tasks may vary depending on the hemisphere and the type of stimuli or strategies involved (Barch et al., 1997; Jonides et al., 1998; Honey et al., 2000).

In Luria’s conception, patients with lesions of the left parietal cortex experience problems related to memory. Although he did not specifically refer to working memory, he did highlight that the mnemonic difficulties were not related to global recognition but rather spatial recognition, such as the inability to memorize operations. In terms of primary or non-specific mnemonic processes, a better conceptualization of working memory has emerged. Patients with deep brain lesions affecting the hippocampus and Papez circuit perform poorly on recognition tests, but the contribution of the parietal cortex was not yet evident in Luria’s work (Luria, 1973).

### 4.7. Organization of the movement

The left parietal lobe is known to participate in the dorsal visual processing pathway, which is responsible for registering spatial position and visually guided action control (Goodale and Milner, 1992). These roles attribute to the left parietal lobe a part in controlling skilled motor actions, which is consistent with studies conducted by various researchers where left parietal lesions are the major cause of limb apraxia (Geschwind, 1975; Heilman et al., 1982; De Renzi et al., 1983; Buxbaum et al., 2007). The left parietal cortex, particularly the supra-marginal gyrus, also has a motor attention function, and when it is compromised, individuals face difficulties in changing the focus of motor attention, which is one of the possible causes of apraxia observed in patients with left parietal lesions (Rushworth et al., 1997).

The formation of movements is linked to a conscious act characterized by motor intention because, during movement, information from the muscular periphery and the retina reaches the cortex, providing objective knowledge of the movement execution (true motor consciousness; Carota et al., 2010). However, recent evidence suggests that the intention to move is conscious, independent of movement execution (Wolpert and Kawato, 1998; Desmurget and Sirigu, 2009; Carota et al., 2010), characterizing a subjective feeling of moving (conscious motor consciousness). This conscious activity of movement intention is associated with increased activity of the posterior parietal cortex (Desmurget and Sirigu, 2009). Studies that applied electrical stimulation in the right inferior parietal cortex showed the desire to move contralateral limbs, while electrical stimulation in the left inferior parietal cortex caused the intention to move the lips and speak. By increasing the stimulation intensity in the parietal areas, participants believed they had performed the desired movements, demonstrating that conscious intention and motor consciousness come from an increase in parietal activity before execution (Desmurget et al., 2009).

It is believed that movement intention and awareness are generated and monitored in the inferior parietal cortex (Desmurget and Sirigu, 2009). Studies conducted by Benjamin Libet et al. showed that the judgment of the intention to move precedes the onset of movement by about 200 milliseconds, while the readiness potential (RP) precedes judgment by about 1 s (Carota et al., 2010). Another study, conducted by Haggard and Eimer (1999), in which patients needed to decide with which hand to respond (left or right), found that the lateralized readiness potential (LRP) occurred 800 milliseconds before the onset of movement (Haggard and Eimer, 1999). The delay resulting from these studies is similar to the time required for reactive movements in response to audiovisual information, suggesting that motor response is processed after motor intention (Desmurget and Sirigu, 2009).

Lesions in the posterior parietal cortex trigger extreme hand movements and loss of conscious motor awareness (Desmurget and Sirigu, 2009), as well as weakness and deficits in motor imagery (Aflalo et al., 2015). Studies by Sirigu et al. (2004) used the Libet paradigm (Carota et al., 2010) in patients with posterior parietal cortex lesions, showing that the delay between the judgment and the start of movement was only 55 milliseconds, while in control individuals, the delay was over 250 milliseconds. These results suggest that patients did not know the intention to move until the movement became imminent (Sirigu et al., 2004), and the patients with lesions did not know which movement they wished to make, suggesting that the posterior parietal cortex contains stored representations of movement (Rothi et al., 1991; Sirigu et al., 1995) and that electrical stimuli activate these representations, causing the desire to change (Desmurget et al., 2009). Thus, the posterior parietal cortex generates a conscious awareness of movement related to motor prediction and selection (Desmurget et al., 2009; Haggard, 2009), as well as being important in the activation and maintenance of final motor intentions (Sirigu et al., 2004).

The parietal cortex is involved in the formation of conscious motor images. When the cortex is damaged, individuals lose the ability to predict through mental simulation and lose the conscious intention to move (Sirigu et al., 2004). These visuomotor transformations occur in the anterior intraparietal area of the posterior parietal cortex, where there are neurons with motor and visual properties (Fogassi and Luppino, 2005). The anterior intraparietal area is related to the ventral premotor cortex in visuomotor transformations for visually oriented manual actions to objects (Jeannerod et al., 1995; Sakata et al., 1997). The area is also connected to the inferotemporal region, which is important for object recognition through pragmatic analysis or identity-related information (Tanaka, 1996). This connection demonstrates that the posterior parietal cortex is an integrator of sensory and motor signals, performing transformations for appropriate motor planning (Fogassi and Luppino, 2005), thus adding motor functions to the parietal lobe that were not predicted by Luria (1973).

To demonstrate motor planning and internal movement image action, Sirigu et al. (1999) evaluated apraxic patients. In the study, patients were subjected to simple and complex movements that were recorded and shown to them along with the examiner’s imitation of the movement. The patients were able to accurately identify their hands on the screen when the examiner’s movement was different from the ones that they performed. When the two movements were congruent, there were problems with discrimination. Another finding that supports the execution of an internal image of the movement is that when patients observed on the screen movements performed by the examiner that imitated the movement caused by an alien hand, they believed that the movement had been correctly executed by them, demonstrating that lesions in the left parietal cortex alter representational aspects of gestures and suggesting a failure in feedback on the movement executed and internal image (Sirigu et al., 1999). This finding is in line with Luria’s concepts, as he believed that a lesion in the left parietal cortex, specifically the occipitoparietal region, led to movement disorders, such as failures in the imitation and comprehension of motor commands (Luria, 1973).

The activity of neurons in the posterior parietal cortex reflects movement plans and can be used to control cursors on computer screens (Musallam et al., 2004; Mulliken et al., 2008; Hauschild et al., 2012). That activity is often utilized in patients affected by tetraplegia through neural prostheses to control external devices by generating imagined movements (Andersen et al., 2014).

Studies conducted by Aflalo et al. implanted microelectrodes in a tetraplegic patient whose complete spinal cord injury occurred at C3-C4. The implantation occurred in 94 areas, both inside and outside the posterior parietal cortex, providing images of cortical activation areas by fMRI. The equipment was connected to a computer, which decoded the impulses and created on the screen images of the movements that the patient was imagining, demonstrating complex and correct movements. This experiment proved the functional properties of a large neuronal population in the posterior parietal cortex as well as the discovery that there are some neurons with high specificity for the left or right limb. This study showed that the posterior parietal cortex is involved not only in motor intention but also in non-motor intention (Aflalo et al., 2015).

Voluntary actions are characterized by conscious intention. It was previously believed that conscious intention emerged in the mesial precentral area, including the supplementary and pre-supplementary motor areas (Haggard, 2005). However, this assumption was challenged by evidence indicating the contribution of the IPL (Fridman et al., 2011). Studies by Desmurget and Sirigu showed that the inferior parietal cortex is involved in the subjective experience of wanting to change before motor planning, while the precentral area carries out the impulse to change with the movement already planned and ready to happen (Desmurget and Sirigu, 2012). It was also discovered that the inferior parietal cortex plays a role in organizing and understanding the intention of action. It is connected to the ventral premotor area and forms the parieto-premotor system. Moreover, it contributes to the fluidity of action execution and the realization of a basic and automatic understanding of motor intention (Bonini et al., 2010).

In Luria’s observations, the parietal cortex did not present motor selection or motor planning functions. However, it is now known that cortical functions are summed, and the parietal cortex has various functions that could not be observed with the technology available at the time (Luria, 1973).

The posterior parietal cortex is responsible for integrating different types of sensory information to produce multiple representations of space combined with actions (Rizzolatti et al., 1997; Snyder, 2000). However, studies on monkeys and humans have highlighted that in addition to motor planning, postural monitoring also occurs (Sirigu et al., 1995; Wolpert et al., 1998). This occurrence dissociates the extrinsic (visual) and intrinsic (postural) coordinates of movement. Studies by Pellijeff et al. used fMRI to investigate the brain areas involved in body schema, elucidating the role of the superior parietal cortex, mainly the precuneus, which, when damaged, leads to errors in reaching visually guided objects (Pellijeff et al., 2006). Considering extrinsic coordinates, Corbetta et al. (2000) demonstrated that activation of the intraparietal sulcus occurs before visual presentation of the target, while the right temporo-parietal junction activates with target detection (Corbetta et al., 2000). These findings complement the results obtained by Luria in his 1973 book (Luria, 1973), in which motor functions and body schema are not portrayed as designations of the parietal lobe.

The parietal cortex is involved in an early stage of motor planning. Studies on monkeys have highlighted that neurons in the intraparietal sulcus are responsive to eye movements, anticipating retinal consequences and coordinating them for a precise representation of visual space (Duhamel et al., 1992). However, it is believed that the posterior parietal cortex presents sub-regions containing intention maps related to the planning of different movements (Andersen and Bueno, 2002). Evidence indicates that the posterior parietal cortex also participates in the detection of incompatibilities between desired and executed movements when visual feedback is important, such as correcting trajectory with target changes (Blakemore and Sirigu, 2003).

Studies conducted by Andersen and Bueno have highlighted the sub-regions currently elucidated in the PPC (Andersen and Bueno, 2002). Specifically, the lateral intraparietal area specializes in planning, the medial intraparietal area encodes movement direction, the anterior intraparietal area is responsible for visuomotor transformations (Andersen and Bueno, 2002), the lateral margin of the intraparietal sulcus is related to peripheral attention and eye movements, the medial margin of the intraparietal sulcus is related to the planning of manual movements (Andersen and Bueno, 2002; Vesia and Crawford, 2012), and the posterior-medial region in the superior parieto-occipital cortex has a function of reaching actions in peripheral locations (Vesia and Crawford, 2012).

Culham and Valyear (2006) demonstrated the existence of sub-regions of the posterior parietal cortex with different functions. Starting with the dorsal stream, in which actions are guided visually, activity was evidenced in preparation for the actions and observation of other people with the perceptual processing of attributes and resources relevant to actions even when none were executed. Furthermore, it was demonstrated that lesions in specific regions of this cortex are related to optic ataxia, motor actions, and eye movements (Culham and Valyear, 2006). Optic ataxia is also related to lesions of the occipito-parietal junction (Karnath and Perenin, 2005), in which coordinated hand movements are impaired by visual problems (Hwang et al., 2012) and can be compared to the description of fixation ataxia, as described by Luria (1973).

According to Luria, the parietal cortex would only have somatosensory functions, not participating in motor actions (neither planning nor execution), including the tertiary zone (inferior parietal cortex and parieto-occipital), which, when damaged, would lead to a disorder in the reception and analysis of information (Luria, 1973). However, it is currently known that the cortex functions in an integrated way, with communication and co-participation from different areas for complex activities.

As previously stated, the parietal lobe presents the somatosensory cortex and the posterior parietal cortex. The PPC is involved in various cognitive processes. Behrmann et al. (2004) observed that selective attention is one of these processes (Coull and Frith, 1998; Coull and Nobre, 1998; Wojciulik and Kanwisher, 1999; Marois et al., 2000a), in which the polarized attention signal can be generated in an oriented way to stimuli (attentional capture) or resulting from the explicit will of the organism (Behrmann et al., 2004). Neuroimaging results infer that attentional capture, independent of modality, arises at the temporoparietal junction (Downar et al., 2001, 2002), while explicit will arises in the superior parietal lobe and precuneus region (Yantis et al., 2002; Giesbrecht et al., 2003; Liu et al., 2003; Yantis and Serences, 2003). However, some studies also suggest the participation of the IPL (Husain and Nachev, 2006).

The parietal cortex plays a crucial role in monitoring the internal and bodily representations of actions, which are differentially specialized. Damage to the left parietal lobe affects the ability to generate and monitor an internal model of the movement to be executed, while lesions in the right parietal lobe result in disturbances in the internal representation of one’s own body (Daprati et al., 2010). It should be noted that patients with bilateral parietal lesions present with apraxic gaze, which is an inability to voluntarily direct one’s gaze to the periphery and optic ataxia (Husain and Stein, 1988; Vallar, 2007), demonstrating alterations in the intentional and attentional aspects of voluntary actions with a loss of bodily awareness and awareness of motor action (Daprati et al., 2010). Luria’s studies (Luria, 1973) had earlier revealed that right parietal lesions resulted in dysfunctions in the body schema; however, as already stated, there was no association with movement or the parietal lobe.

Studies conducted by Wolpert et al. (1998) postulated that the parietal cortex is also involved in maintaining and updating the bodily state emitted from sensory and motor signals. This conclusion was reached by analyzing patients with parietal lobe lesions who reported the disappearance of their limbs both sensorially and in a motor sense (Wolpert et al., 1998).

In his book The Working Brain Luria explained that patients with lesions in the right parietal lobe had an unawareness of the left half of the visual field, whether of their own body or the environment as a whole (Luria, 1973). This suggests that in Luria’s conceptions, the parietal lobe was essential for the formation of the sensory image of the body and the reception of stimuli.

### 4.8. The neo-Lurian approach and epilepsy

A neo-Lurian approach utilizes Luria’s functional units as the foundation for developing the concept of PASS, which consists of four major cognitive processes: Planning, Arousal-Attention, and Simultaneous and Successive processing (Das, 1999). The parietal lobe in general plays a significant role in the second functional unit, which is associated with memory processes (Luria, 1973; Aversi-Ferreira et al., 2010; Sugahara et al., 2021), while the posterior-inferior part of the parietal lobe is involved in non-modal functions related to the third functional unit (Sugahara et al., 2021), and the postcentral gyrus being a modal sensory area.

A systemic theory of the brain, as proposed by Luria, provides a framework for understanding the complex relationship between seizures and cognitive function. Although he did not focus specifically on epilepsy, his theory that brain damage can disrupt dynamic cognitive processes, even if the damaged brain region is not directly involved in those processes, aligns well with the findings of modern neuroimaging and clinical studies (Patrikelis et al., 2017; Traianou et al., 2019). This is because different brain regions are interconnected, and damage to one region can have cascading effects on other regions. For instance, the ictal psychic manifestations of epilepsy could be explained by episodic changes in the body schema associated with the parietal lobe (Luria, 1973; Patrikelis et al., 2017).

Another example is parietal lobe epilepsy (PLE), a rare type of focal epilepsy that can cause somatosensory symptoms, paresthesia, deficiencies in body perception, sense of burning, vertigo, mood changes, gustatory hallucinations, and rarely aphasia, alexia, dyscalculia, and hemineglect (Traianou et al., 2019). These PLE symptoms corroborate Luria’s model, which views brain functions as working dynamically and interacting nonlinearly in the space–time process (Zaytseva et al., 2015). This means that damage in one area of the brain may generate cognitive symptoms functionally associated with other areas, even when these other areas are not directly affected. This contrasts with the traditional view of superior mental functions being located in specific anatomical regions.

Evidence from epilepsy validates the idea of a syndromic view, which is based on the observation that neurological disorders often produce a constellation of symptoms that are not all directly related to the site of the brain lesion. As previously mentioned, Luria did not specifically mention epilepsy, but the same concept could be similarly observable in the case of anomia as directly described by him. As higher psychological functions are distributed across a network of isolated functions, syndromes may appear when some disarrangement occurs in a part of the system, being characterized by a cluster of related symptoms which may reflect the disruption of different psychological processes. Therefore, anomia could arise by lesions in different, apparently unrelated, brain areas, such as memory areas, the secondary listening area in the temporal lobe, or visual representation areas in the posterior temporal cortex (Luria, 1973; Aversi-Ferreira et al., 2019).

While this work is not specifically about neo-Lurian aspects, these points emphasize the view that Luria’s work deserves to be considered in modern analyses of the neuropsychological aspects of the mind and its functions. The observation of Luria’s methodology and theory about lesions could be applied to explain cognitive processes and pathologies of the brain.

## 5. Conclusion

The human cortical structure is the most complex structure on the evolutionary scale. The structure’s association areas are the most complex in terms of connections and associations between layers, with a predominance of layers II and III, responsible for the integration of various stimuli, participating in cognitive activities, and simultaneous synthesis, as cited by Luria in the cytoarchitecture analysis of the cortical parietal tissue.

The neuropsychological aspects involved in the parietal cortex are varied, depending on the hemisphere involved and the affected region. The diversity is also due to the neural connections with several cortical and subcortical regions, which can be idiosyncratically modulated due to the lateralization of the functions caused by cortical dominance.

The main neuropsychological deficits associated with the lesioned parietal lobe are apraxia, dyscalculia, and language; deficiencies in activities that involve manual tasks and the hand alien syndrome; negligence and attention; and memory and movement planning. Moreover, modern discoveries are similar to Luria’s studies for apraxia, including pantomime.

Problems related to dyscalculia are similar between Luria’s studies and more recent ones, as far as language is concerned. However, Luria’s studies on language need to be analyzed separately because the theories of socio-cultural-historical psychology involve development beyond just brain structures, taking into account the environment and human evolution. In this sense, Vygotsky and Luria anticipated the modern view of language evolution (Aversi-Ferreira et al., 2021).

Regarding all the topics discussed in this article, modern studies have discovered findings similar to those of Luria’s studies indicating that the neuropsychological method he used in lesion analysis deserves high consideration in the scientific community and that his studies have been neglected in more recent scholarship. Indeed, the neurophysiological basis for brain function found in The Working Brain: An Introduction to Neuropsychology needs to be considered in neural studies and included in didactic books. An exception to the similarities among modern studies was regarding the type of memories that Luria considered differently in terms of concepts and names compared to the modern approach.

In conclusion, the human cortical structure, particularly the parietal cortex, is a complex and dynamic region responsible for a range of neuropsychological functions. Luria’s studies on the cytoarchitecture and function of the parietal cortex are still highly relevant today, with modern studies supporting many of his findings. The neuropsychological deficits associated with lesions to the parietal lobe, such as apraxia, dyscalculia, and language impairments, continue to be areas of research interest. However, further studies are needed to better understand the mechanisms and connections involved in these pathologies, particularly with the use of modern imaging techniques. It is essential that Luria’s contribution to the understanding of the neuropsychology of the parietal lobe continue to be recognized and included in didactic books, as his work laid the foundation for current research in this area.

## Data Availability Statement

The original contributions presented in the study are included in the article only, further inquiries can be directed to the corresponding author.

## Author contributions

All authors listed have made a substantial, direct, and intellectual contribution to the work, and approved it for publication.
